# Training high-strength aluminum alloys to withstand fatigue

**DOI:** 10.1038/s41467-020-19071-7

**Published:** 2020-10-15

**Authors:** Qi Zhang, Yuman Zhu, Xiang Gao, Yuxiang Wu, Christopher Hutchinson

**Affiliations:** Department of Materials Science and Engineering, Monash University, Clayton, 3800 VIC Australia

**Keywords:** Mechanical properties, Metals and alloys

## Abstract

The fatigue performance of high strength aluminum alloys used in planes, trains, trucks and automobiles is notoriously poor. Engineers must design around this important limitation to use Al alloys for light-weighting of transportation structures. An alternative concept for microstructure design for improved fatigue strength is demonstrated in this work. Microstructures are designed to exploit the mechanical energy imparted during the initial cycles of fatigue to dynamically heal the inherent weak points in the microstructure. The fatigue life of the highest strength Aluminum alloys is improved by 25x, and the fatigue strength is raised to ~1/2 the tensile strength. The approach embraces the difference between static and dynamic loading and represents a conceptual change in microstructural design for fatigue.

## Abstract (web summary)

The fatigue performance of high strength aluminum alloys is notoriously poor, leading to design limitations for transportation structures. Here the authors design microstructures to exploit the mechanical energy of fatigue to dynamically heal the microstructural weak points and improve the high cycle fatigue life.

## Introduction

Aluminum (Al) alloys are the second most popular engineering alloy in use today. Compared to steel, they are light (1/3^rd^ the density of steel), non-magnetic and have excellent corrosion resistance. The precipitate-strengthened Al alloys can also be processed to be relatively strong so their specific mechanical properties (property/density) provide a competitive advantage in applications where weight reduction is important. An example is transport applications, such as planes, trains, trucks, and automobiles. The transportation industry emphasizes weight reduction to reduce fuel emissions, and this has led to the continual increase in use of Al alloys in these important applications^1–3^.

Transportation structures are subject to alternating forces and the stresses the materials must withstand are cyclic in nature. This loading leads to fatigue^4–9^ and the resistance of a material to fatigue failure is critical in these applications. It is estimated that 80% of all engineering alloy failures are due to fatigue^5,8^. The cyclic stress that an alloy can support for a prolonged period (~10^7^ cycles) without failure is known as the fatigue strength, and it is always lower than the tensile stress that would lead to failure under monotonic loading. The fatigue strength (a dynamic property) and tensile strength (a static property) are strongly correlated in the case of steels: $fatigue\;strength/tensile\;strength\sim 1/2$ (Supplementary Fig. 1)^10^. This highlights one strategy taken when an improved fatigue strength is required—a material with a higher tensile strength is chosen.

**Fig. 1:**
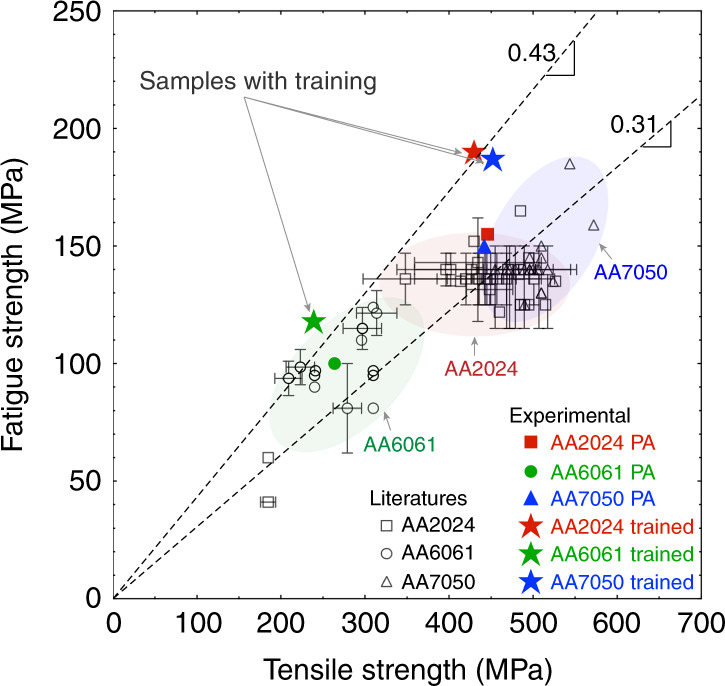
The fatigue strength vs. ultimate tensile strength (UTS) for commercial AA2024, AA7050, and AA6061 alloys. Correlation between fatigue strength and tensile strength for three Al alloys.^10–15^.

Unfortunately, the fatigue performance of high strength Al alloys is comparatively poor, and this is one of the Achilles heels of Al alloys. Figure 1 shows the fatigue and the tensile strength correlation for three of the most common precipitate-strengthened Al alloys: AA2024 (Al–Cu–Mg), AA6061 (Al–Mg–Si), and AA7050 (Al–Zn–Mg–(Cu)). The fatigue strength of Al alloys is ~1/3 of their tensile strength. Engineers are forced to design around this limitation when using high strength Al alloys in applications where fatigue is a limiting property. Despite the efforts of the materials scientist to modify the microstructure of Al alloys to make them stronger, the corresponding improvement in the fatigue strength is much less than it would be for a steel.

Fatigue failure occurs in stages. Cyclic loading leads to microplasticity and the accumulation of irreversible damage in the form of a localization of plasticity (usually associated with defects). The plastic localization catalyzes the initiation of a fatigue crack. The fatigue crack grows and leads to final fracture^5,16^. All the stages are important but the relative contribution to the overall fatigue lifetime depends on the external loading conditions. In high cycle fatigue (HCF), where the cyclic stress is significantly lower than the monotonic yield strength, most of the lifetime is consumed by plasticity localization and the initiation of a critically sized fatigue crack^6,17,18^. In many (but not all) transport applications, the cycles of alternating stress fall in the HCF regime and therefore it is the HCF performance which is of interest. An important exception is aircraft, where the low-cycle fatigue (LCF) performance is of particular interest.

In this report, we introduce an alternative conceptual approach to substantially improve the HCF performance of precipitate-strengthened Al alloys. We demonstrate increases in the fatigue lifetime of an order of magnitude or more, and increases in the fatigue strength to ~1/2 of the tensile strength, as is the case for steels. The approach embraces the differences between static and dynamic loading and exploits the imparted mechanical energy associated with the initial cycles of fatigue to modify the microstructure in a way that resists the localization of plasticity and significantly increases the time to initiate a fatigue crack. It is a form of self-healing, or training, and represents a conceptual change in microstructural design of precipitate strengthened materials for HCF performance.

## Results

### High cycle fatigue response of precipitate-strengthened aluminum alloys

The precipitate-strengthened Al alloys obtain their high strength from a fine distribution of nanoscale particles that form by a nucleation and growth process during elevated temperature processing. The precipitation process has been well studied and treatments have been designed to obtain the highest strength states (i.e. peak aged state—PA). Commercially available AA2024, AA6061, and AA7050 alloys are used in this work. These materials have been processed using standard approaches (Methods section and Table S1) and prepared in both the peak aged state (PA) as well as an under aged state (UA). The precipitates in the UA state are smaller, and the volume fraction reduced, compared to the PA state. The UA heat treatment times are chosen to give a hardening increment approximately half the total hardening increment observed during the PA treatment. The precipitate microstructures of the UA and PA states of the three alloys, as well as the room temperature monotonic tensile behavior are shown in Supplementary Figs. 2 and 3. The yield strength (*σ*_y_), ultimate tensile strength (UTS), and uniform elongation (*ε*_u_) are summarized in Table 1. The yield and tensile strengths of the UA state are lower than the PA state for each alloy.

**Table 1:** The yield strength (*σ*_y_), ultimate tensile strength (UTS), and uniform tensile elongation (*ε*_u_) of the under aged (UA) and peak aged (PA) AA2024, AA6061, and AA7050 alloys.

Alloy	Conditions	*σ*_y_ (MPa)	UTS (MPa)	*ε*_u_
AA2024	UA	215 ± 4	428 ± 9	0.19 ± 0.01
	PA	296 ± 4	446 ± 15	0.09 ± 0.02
AA6061	UA	104 ± 9	210 ± 7	0.20 ± 0.04
	PA	221 ± 7	264 ± 9	0.07 ± 0.01
AA7050	UA	227 ± 8	403 ± 10	0.22 ± 0.03
	PA	317 ± 7	442 ± 10	0.13 ± 0.03

**Fig. 2:**
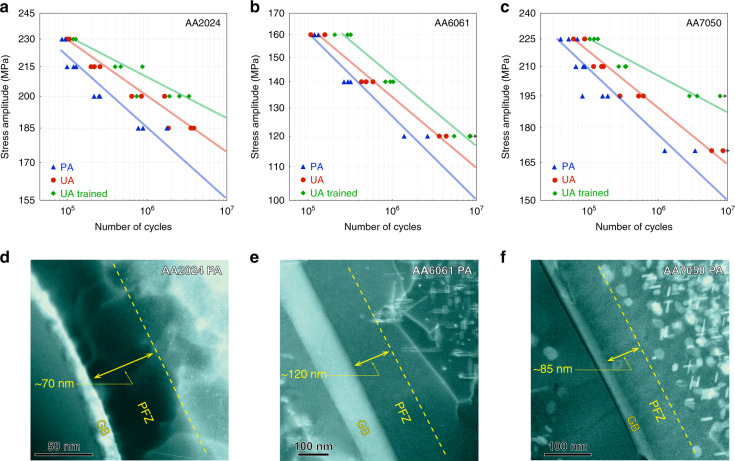
High cycle fatigue (HCF) S-N curves and precipitate-free zones (PFZ’s) of the under aged (UA), peak aged (PA), and trained alloys. **a**–**c** HCF S-N curves of under aged (UA), peak aged (PA), and trained AA2024 (**a**), AA6061 (**b**), and AA7050 (**c**) alloys. The HCF tests were fully reversed (*R* = −1) at a frequency of 20 Hz. **d**–**f** LAADF-STEM images show PFZ’s formed in the PA alloys. The electron beam direction was parallel to <100>_Al_ in **d**, **e**, and parallel to <110>_Al_ in **f**.

The HCF life of these alloys have been measured using fully reversed (*R* = −1) fatigue loading of smooth samples and the results are shown in Fig. 2a–c. Where the samples ran-out to 10^7^ cycles (e.g. PA AA7050), that stress level is taken as the fatigue strength. For the AA2024 and AA6061, the S-N curves were extrapolated to 10^7^ cycles to obtain an estimate of the fatigue strength. The experimentally measured fatigue strength of the PA samples has been added to Fig. 1 and is consistent with the values reported in the literature. The key observation in Fig. 2a–c is the weaker UA state has consistently better HCF performance at all investigated stress levels than the stronger PA state. These observations are the opposite of conventional wisdom based on the correlation between the fatigue strength and tensile strength of steels (Supplementary Fig. 1).

The origin of the difference in fatigue performance between the UA and PA states can be due either to differences in the time required to initiate a fatigue crack, or differences in the fatigue crack growth (FCG) behavior. The FCG has been measured for each alloy in both the UA and PA states and is shown in Supplementary Fig. 4. The FCG has been measured both parallel and perpendicular to the rolling direction (RD). There is no significant difference between the FCG behavior of the UA and PA states for the same FCG orientation. As is usually the case for HCF testing of smooth samples, the differences in lifetime are dominated by differences in the time to initiate a critically sized fatigue crack.

### Localization of plasticity in precipitate-free zones

A key feature of precipitate-strengthened Al alloys is the presence of precipitate-free zones (PFZ’s) adjacent to grain boundaries. The nucleation and growth of precipitates is mediated by vacancy diffusion and is therefore sensitive to the concentration of vacancies^19,20^. During thermal processing, excess vacancies annihilate at grain boundaries leaving a region depleted in vacancies. Solute is less mobile in this region and precipitation is retarded. With further aging at elevated temperatures, these regions also become depleted in solute due to heterogeneous precipitation on grain boundaries^19–21^. The result is a layer of material adjacent to grain boundaries that is precipitate-free and comparatively soft compared to the precipitate-strengthened grain interiors. The PFZ’s for the alloys examined in this study are shown in the low-angle annular dark-field (LAADF) scanning transmission electron microscopy (STEM) images in Fig. 2d–f.

**Fig. 3:**
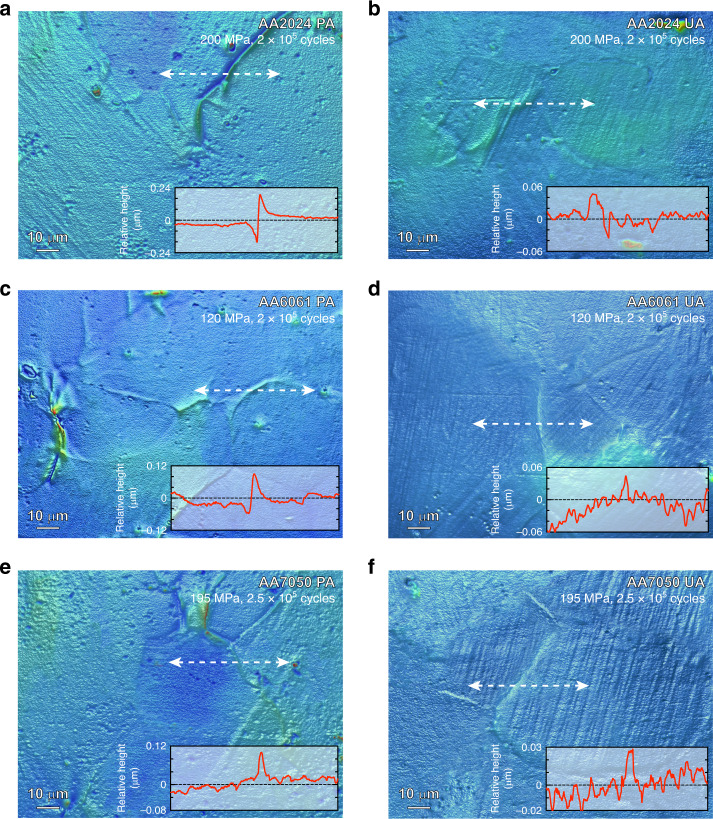
Surface evolution of air quenched (AQ) AA2024, AA7050, and AA6061 alloys with obvious precipitate-free zones (PFZ’s). **a**, **c**, **e** The large localized surface relief along the grain boundaries for the peak aged (PA) alloys after certain number of high cycle fatigue (HCF). **b**, **d**, **f** The uniform surface relief formed through the grains for the under aged (UA) alloys after certain number of high cycle fatigue (HCF). The red curves show the relative height of different surface relief highlighted by white arrows.

During fatigue loading, plasticity becomes localized in these soft PFZ’s and the irreversible cyclic dislocation motion leads to an accumulation of damage that provides the conditions for fatigue crack initiation. During HCF, cracks almost always initiate on the surface of the material and the evolution of an initially smooth surface during cyclic loading provides a signature of the localization of plasticity that leads to crack initiation. Figure 3a, c, e demonstrate optical profilometry images of the external surfaces of the PA AA2024, AA6061, and AA7050 materials after ~10^5^ cycles of loading. The localization of plasticity in the PFZ’s is clearly seen by the generation of extrusions and intrusions of material adjacent to grain boundaries with heights of 100–150 nm. This plastic localization grows during cycling and has been quantified for a large number of grains in Supplementary Fig. 5. It is this plastic localization that leads to fatigue crack initiation. In contrast, the optical profilometry images of the UA states, Fig. 3b, d, f, subject to the same number of cycles at the same stress levels, exhibit much less localization of plasticity. Rather, the plasticity is more uniformly distributed throughout the grains and the irreversible changes to the surface roughness are only ~20–40 nm in height. They also evolve more slowly during cycling than the localization at grain boundaries in the PA states (Supplementary Fig. 5). It is this greater homogeneity in plasticity of the UA samples, compared to the PA states, that delays the initiation of a critically sized fatigue crack and leads to the greater fatigue life of the UA state, even though the UA materials have lower yield and tensile strengths than the PA state (Table 1).

## Discussion

### Dynamic precipitation in PFZ’s

The UA and PA precipitate-strengthened Al alloys both contain PFZ’s but they behave differently during high cycle fatigue loading. Despite looking superficially the same, the PFZ’s in the UA and PA states are not exactly the same. During the early stages of the precipitation process, the PFZ’s are vacancy depleted. They contain solute, but precipitation does not occur for kinetic reasons. This is the case for the UA microstructure. As the precipitation process proceeds, the PFZ’s also become depleted in solute due to the solute diffusion to the grain boundary to form grain boundary precipitates. This is the case for the PA microstructure. The presence, or not, of a supersaturation of solute within the PFZ’s has profound implications for the response of the PFZ to cyclic plasticity. Recent work has shown that dynamic precipitation can be induced to take place in Al alloys at room temperature when subject to cyclic plasticity^22–25^. The back-and-forth motion of dislocations leads to the generation of vacancies (due to dragging of dislocation jogs) and this injection of vacancies can mediate diffusion and precipitation, even at room temperature. It leads to a pronounced strengthening effect^22^.

**Fig. 4:**
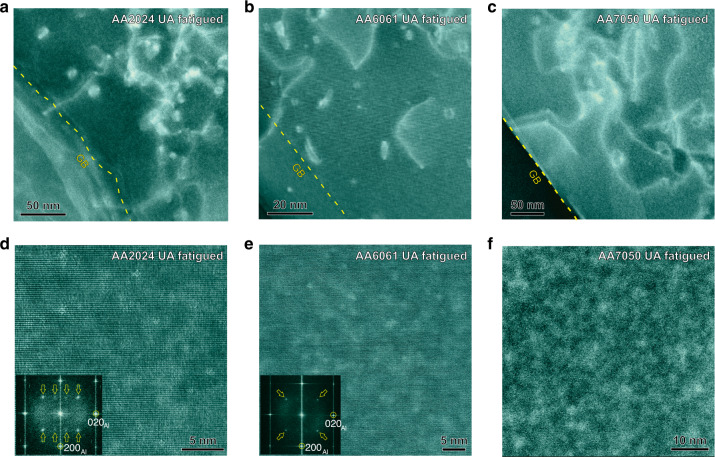
LAADF-STEM images showing the microstructure of precipitate-free zones (PFZ’s) after HCF deformation. **a**–**c** PFZ’s of high cycled fatigue (HCF) treated under aged (UA) samples for AA2024 (**a**), AA6061 (**b**), and AA7050 (**c**). AA2024 was fatigued at 185 MPa for 3.5 × 10^6^ cycles, AA6061 was fatigued at 120 MPa for 4 × 10^6^ cycles, and AA7050 was fatigued at 175 MPa for 8 × 10^6^ cycles. **d**–**f** High-magnification LAADF-STEM images showing nanoprecipitates existing in PFZ’s pointed out in **a**–**c**. Corresponding fast Fourier transform (FFT) patterns inserted in **d**, **e** reveal diffraction from nanoprecipitates (pointed by yellow color hollow arrows). The electron beam direction was parallel to <100>_Al_ in **a**, **b**, **d**, **e** and parallel to <110>_Al_ in **c**, **f**.

The PFZ’s of the UA material contain solute. During the initial cycles of fatigue, microplasticity is localized in the soft PFZ’s, but the dislocation motion generates vacancies that promote dynamic precipitation and strengthen the PFZ. The removal of the strength differential between the grain interior and the original PFZ, leads to a greater homogeneity of plasticity throughout the material (Fig. 3b, d, f). The dynamic precipitation in the PFZ’s can be seen in the LAADF-STEM images for each alloy in the UA state after cycling in Fig. 4. The dynamically formed precipitates are of the order of 1 nm in size and particles of this size provide a major strengthening increment^22^. Plasticity is also localized in the PFZ’s of the PA samples during cycling (Fig. 3a, c, e) but because they are both vacancy and solute depleted, the generation of vacancies due to dislocation motion does not have solute available to facilitate dynamic precipitation and hence they are not strengthened. No dynamic precipitation is observed in the PFZ’s of cycled PA materials (Supplementary Fig. 6).

### Training for improved high cycle fatigue behavior

The observations in Fig. 2a–c that the weaker UA materials have better HCF performance than the stronger PA materials is because the PFZ’s in the UA materials can dynamically strengthen due to dynamic precipitation in the early cycles of fatigue and this enhances the resistance to plastic localization and fatigue crack initiation. With this understanding, we may now consider an alternative approach to designing Al alloys for fatigue performance. Instead of using a traditional PA material, we may instead start with an UA material and design a specific cyclic training scheme to repair the PFZ’s by dynamic precipitation to decrease the strength differential between the grain interiors and the PFZ’s. We should expect a significantly greater fatigue life.

**Fig. 5:**
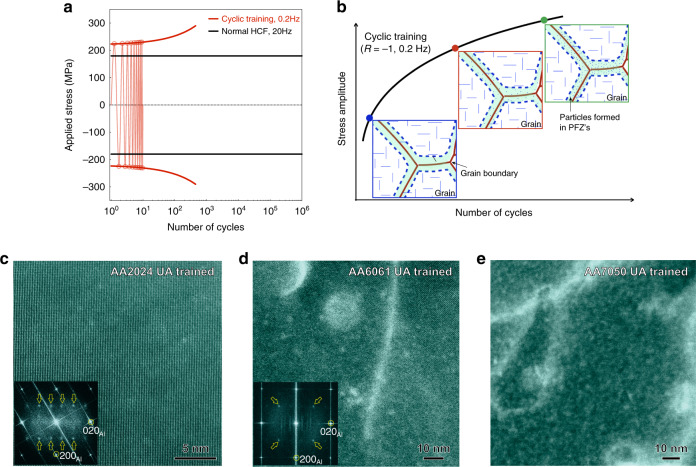
Cyclic training and microstructure evolution during cyclic training for the under aged (UA) alloys. **a** Schematic illustration of the fully reversed (*R* = −1) cyclic training at 0.2 Hz. **b** Schematic illustration of the precipitation in PFZ’s as the training cycle number increases. **c**–**e** LAADF-STEM images showing nanoprecipitates formed in PFZ’s of AA2024 trained for 450 cycles (**c**), AA6061 trained for 700 cycles (**d**), and AA7050 trained for 450 cycles (**e**). Inset FFT patterns in **c**, **d** show diffraction from nanoprecipitates (indicated by yellow color hollow arrows). The electron beam direction was parallel to <100>_Al_ in **c**, **d** and parallel to <110>_Al_ in **e**.

The formation rate of new particles (N) due to dynamic precipitation during cyclic plasticity has been shown to be linear in plastic strain (ε) (d*N*/d*ε* = const)^23^. Bearing in mind that the formation of new particles leads to strengthening, a cyclic training schedule can be defined to mimic a constant plastic strain amplitude loading as a means to repair the PFZ’s by particle strengthening before the material is subjected to its normal HCF. An example is shown in Fig. 5a. The training schedule is applied at a higher stress than the conventional HCF test. This training is typically several hundred cycles and they are summarized in Supplementary Fig. 7 for the three alloys considered here.

The dynamic precipitation within the PFZ’s of each of the UA alloys after the training process (Fig. 5b), has been examined using LAADF-STEM and is shown in Fig. 5c–e. Some dislocation loops can also be seen and these form as a result of the condensation of excess vacancies generated by the cyclic dislocation motion in the PFZ’s^22^.

The interior of the grains after cyclic training is also shown in Supplementary Fig. 2g–i. As expected, the microstructures resemble the UA state but with the addition of some dislocation loops that form as a result of the condensation of vacancies that form during the cycling. LAADF-STEM images of the grain interiors are also shown at higher magnification in Supplementary Fig. 8. At this higher magnification, additional fine particles can be seen and it is these that cause the enhanced yield and tensile strengths after training (Supplementary Fig. 3).

After the training, the HCF performance of these materials was measured. This data is shown in Fig. 2a–c. In all cases, improvements in the fatigue life are observed at all stress levels. The fatigue life of the AA2024 alloy after training is improved by an order of magnitude compared to the traditional PA state loaded at 200 MPa. The lifetime improvement is a factor of 25x for the trained AA7050 compared to the PA state cyclically loaded at similar stress levels. The fatigue strength of the trained materials has been added to Fig. 1 and now approaches ~1/2 of the tensile strength. The fatigue performance is most improved for the strongest materials (7xxx) and the improvement is smaller for the weakest alloys (6xxx). The reason is that the strength differential between the original PFZ’s and the grain interior is largest for the strongest alloys and these materials suffer most from the strain partitioning to the weaker PFZ’s during fatigue of the PA states. It is the strongest Al alloys that have the most to gain from this alternative approach to microstructure design for fatigue.

High-strength Al alloys have notoriously poor fatigue strength and this has been known for 50 years. In this alternative approach, we embrace the differences between static and dynamic loading and use the mechanical energy imparted into the materials during the early cycles of fatigue to strengthen the weak points in the microstructure (the PFZ’s) by using the early cycles of strain partitioning to drive dynamic precipitation. This strongly delays the localization of plasticity and the initiation of fatigue cracks and leads to enhanced fatigue lives and fatigue strengths. One can imagine how this training schedule could be incorporated into the shakedown tests of structures that use these materials.

Not all precipitate hardened materials contain PFZ’s as damaging as what is typically observed in Al alloys. In alloys where PFZ’s do not exist, or are very small, we would not expect to see an obvious benefit of the training shown in this study. The sizes of PFZ’s in alloys also depend strongly on processing and they are known to be much wider for slow cooling from the solution treatment temperature. The wider the PFZ, the greater the benefit one should expect from applying a training procedure like that introduced in this contribution. There are also limitations to the training procedure shown here—the training must be designed and applied carefully to drive dynamic precipitation in the PFZ to strengthen these weak points, but not lead to catastrophic strain localization in the PFZ in the process. The latter, which could result from a poorly controlled training, could lead to a reduced fatigue lifetime.

The approach outlined in this contribution represents a conceptual change in microstructural design. Instead of designing a strong microstructure and hoping it remains stable for as long as possible during fatigue loading, we recognize that the microstructure will be changed by the dynamic loading and design a starting microstructure (that may have lower static strength) that will change in such a way that its fatigue performance is significantly improved. In this respect, the structure is trained and the training schedule is used to heal the PFZ’s that would otherwise represent the weak points. The approach is general and could be applied to other precipitate hardened alloys containing PFZ’s for whom fatigue performance is an important consideration.

## Methods

### Materials

Commercially available AA2024, AA6061, and AA7050 alloys were used in this study. The compositions and heat treatment conditions are listed in Supplementary Table 1. The alloys were purchased from Kaiser Aluminum Corporation in both extruded rod and plate form. The extruded rods were used for tensile and fatigue samples. Samples from the plates were used for fatigue crack growth (FCG) tests. Solution treatments were performed in a salt bath, followed by air quench to mimic the industrial cooling process, and further aging treatments were conducted in an oil bath. All samples were stored in a freezer at −35 °C after heat treatment to minimize natural aging effects.

### Mechanical testing

The monotonic tensile properties of all alloys were measured using an Instron 4505 machine with a 10-kN load cell and a strain-rate of 0.02 mm/s. A 10-mm extensometer was used.

The high cycle fatigue (HCF) tests were performed using an MTS 858 servo-hydraulic fatigue machine with a 25-kN load frame and a 15-kN actuator combined with a 15-kN load cell. Hour-glass shaped samples were used for HCF and the size was designed according to ASTM standard E466-15 ^26^. The samples have a gauge diameter of 5 mm at the center with the taper radius of 40 mm. The machined surface was manually ground and then polished to surface finish by ~0.04 μm active oxide polishing (OP-S) suspension (colloidal silica suspension). Fully reversed (stress ratio *R* = −1), room temperature high cycle fatigue at 20 Hz was performed for all alloys at different stress levels for all aging conditions. The tests were run for at least 10^6^ cycles and the S-N curves were extrapolated to 10^7^ cycles to estimate the fatigue strength. These extrapolations represent a lower limit for the fatigue strength and are therefore conservative estimates.

Fatigue crack growth (FCG) tests were performed with a NanoBiss 25 kN high precision servo-control fatigue machine using the compliance method with 6-mm-thick compact tension (CT) samples based on ASTM standard E647-15e1 ^27^. Tests were performed using a constant amplitude sinusoidal loading with a load ratio of *R* = 0.1 at a maximum load of 5 kN and a frequency of 5 Hz. The crack length was measured from a Crack Opening Displacement (COD) gauge.

The cycle ‘training’ process of under aged (UA) Al alloys was performed on the MTS 858 servo-hydraulic fatigue machine and the process used stress controlled fully reversed (*R* = −1) cyclic loading at a rate of 0.2 Hz. After training, the samples were re-machined into the hour-glass shape used for all HCF tests and the gauge surface for HCF testing was also manually ground and then polished to surface finish by ~0.04 μm active oxide polishing (OP-S) suspension (colloidal silica suspension). This is necessary to ensure the geometry and surface finish of the trained HCF samples is identical to the HCF UA and PA samples with which they are being compared. The design of the stress controlled training schedule with progressively increasing stress magnitude is based on the cyclic strengthening response during constant plastic strain controlled loading. In the stress controlled training schedule, the square of the average absolute stress increment (Δσ)^2^ is proportional to the cumulative plastic strain (4 N*ε*_p_/2) informed from constant plastic strain controlled cycling^23^. The square of stress increment (Δ*σ*)^2^ is a function of number of cycles (*N*) by:

1 $$({\Delta} \sigma )^2 = (\sigma _i - \sigma _0)^2 = k \cdot N_i$$

where *σ*_i_ is the stress level at cycle number *N*_i_ and *σ*_0_ is the initial applied stress. The constant *k* can be defined through the designed initial stress (*σ*_0_), final stress (*σ*_f_), and number of cycles (*N*):

2 $$k = (\sigma _f - \sigma _0)^2/N$$

The applied stress at any number of cycles can then be calculated to define the cyclic training profile:

3 $$\sigma _i = \sigma _0 + \sqrt {kN_i} = \sigma _0 + \frac{{\sigma _f - \sigma _0}}{{\sqrt N }}\sqrt {N_i}$$

The training process for the UA samples is designed to develop the dynamic precipitation by applying the increasing external stress that is most compatible with the strength increment introduced by dynamic precipitation. This evolving applied training stress effectively limits the introduction of strain hardening due to dislocations. A main criterion for the determination of applied training stress is to regulate the stress magnitude increase from the yield stress of the UA state (*σ*_0_) to a final stress close to the yield stress of PA state (*σ*_f_), based on the assumption that the precipitation hardening increment by dynamic precipitation dominates the strength increase. We have seen that this assumption works well for AA2024 and AA7050 alloys and by cyclic training for a few hundred cycles the yield strength of the trained samples reach the yield strength of the PA condition without introducing significant macroscopic plastic strain. Since the AA2024, AA6061, and AA7050 alloys reach different PA strengths due to their different compositions, the exact shape of their cyclic hardening profiles are also different. Special attention was paid to the AA6061 alloy where the strength increment by cyclic training is comparatively smaller, and we have limited the maximum training stress below the yield stress of peak aged AA6061 to ensure minimal macroscopic strain is introduced during training. From Fig. S9, when comparing the XRD measured microstrains in the samples cycled to the final training stress (*σ*_f_), with the samples monotonically strained to the final training stress (i.e. purely strain hardened), the microstrains in the cycled samples are much smaller. Even though a small amount of plasticity may be introduced by the cyclic training process, the cyclically trained Al alloys are fundamentally different to those strengthened predominantly by strain hardening.

### Microstructural characterization

An FEI Tecnai G^2^ T20 Twin LaB_6_ transmission electron microscopy (TEM) operated at 200 kV was used to characterize the microstructures of the alloys. The low-angle annular dark-field scanning transmission electron microscopy (LAADF-STEM) images were collected on a double Cs-corrected FEI 80-300 Titan^3^ operated at 300 kV in the (semi-) angle range of 19–33 mrad. This imaging mode is based on strain contrast and applied for detailed characterization of small features, such as dislocations or clusters within PFZ’s. The (S)TEM foils were sectioned from both fatigue sample head and sample gauge using a low speed saw with ~0.5 mm in thickness and punched into disks with 3 mm in diameter. The disks were ground to ~0.15 mm thick and twin-jet electropolished in a solution of 33% nitric acid and 67% methanol at −25 °C with 0.2 A current. All foils were cleaned by a Gatan 950 Solarus Advanced Plasma System before observation.

A Veeco NT1100 optical profilometer (OP) was used to measure the surface roughness evolution during fatigue. Vertical scanning interferometry (VSI) mode with a vertical resolution of ~3 nm and lateral resolution of ~0.5 μm was chosen for the surface relief characterization. The samples for surface observation had cylindrical tensile sample geometry with a gauge length of 15 mm and gauge diameter of 5 mm, and a small flat section (10mm long and 2 mm wide) was wire cut at the center of sample gauge. This flat surface was manually ground by sandpaper and then polished with surface finish by ~0.04 μm OP-S suspension.

X-ray powder diffraction (XRD) was used to compare the microstrains between cyclic trained samples and samples monotonically tensile to the same final training stresses. The data were collected on a Bruker D8 Advance diffractometer with Cu Ka radiation and a Lynxeye position sensitive detector. The samples surfaces were finished with OP-S suspension. The XRD data were analyzed using whole-pattern Pawley method as embodied in the software package TOPAS (version 5, Bruker AXS).

## Supplementary information

Supplementary Materials

## Data Availability

The data that support the findings of this study are available from the corresponding author upon request.
