# Supplementary material for: Training high-strength aluminum alloys to withstand fatigue
Source: Nat Commun. 2020 Oct 15;11:5198. doi: 10.1038/s41467-020-19071-7 (PMC7566492; doi:10.1038/s41467-020-19071-7)
Supplement: Supplementary file 1 — Supplementary Materials [file 41467_2020_19071_MOESM1_ESM.pdf]

Supplementary Information

Training high-strength aluminum alloys to withstand fatigue

Qi Zhang, Yuman Zhu, Xiang Gao, Yuxiang Wu, Christopher Hutchinson*

Department of Materials Science and Engineering, Monash University, Clayton, 3800, VIC, AUSTRALIA.

*Correspondence to: christopher.hutchinson@monash.edu

Table S1. Alloys compositions and heat treatment conditions for AA2024, AA6061 and AA7050.

Alloy	Compositions (wt.%)	Solution treatment	Aging treatment	
			UA	PA
AA2024	Al-4.6Cu-1.3Mg-(Zn-Mn-Si-Fe-Cr-Ti)	493°C 1h, air quenching	191°C 30min	191°C 7h
AA6061	Al-1Mg-0.67Si-(Cu- Zn-Mn-Fe-Cr-Ti)	530°C 1h, air quenching	177°C 20min	177°C 20h
AA7050	Al-2.2Cu-2.3Mg-6.2Zn-(Mn-Si-Fe-Cr-Ti)	480°C 1h, air quenching	150°C 10min	150°C 10h

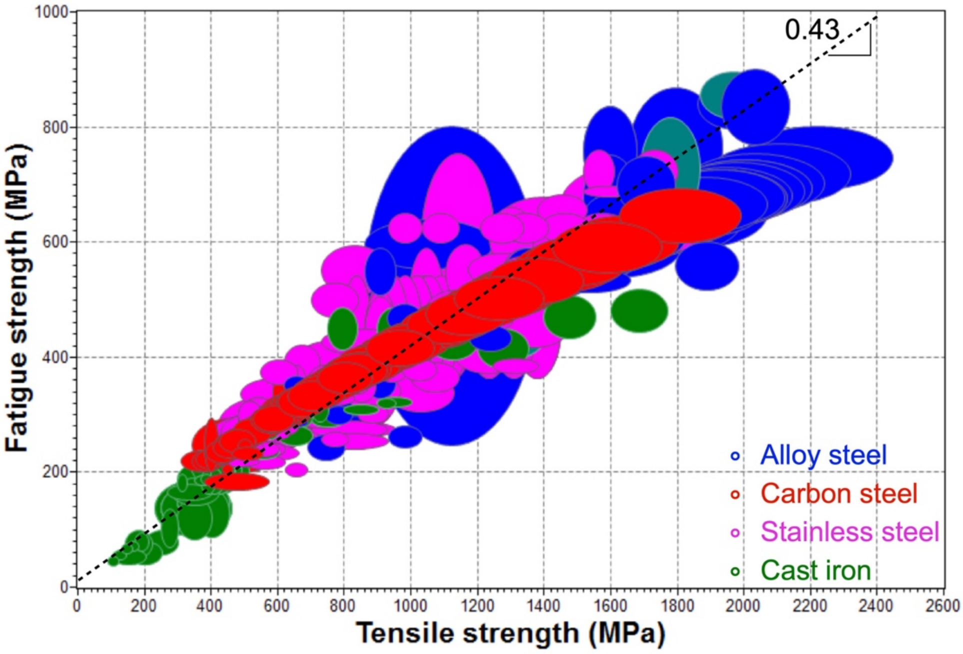

Fig. S1. Fatigue strength versus tensile strength for alloy steel, carbon steel, stainless steel and cast iron [10].

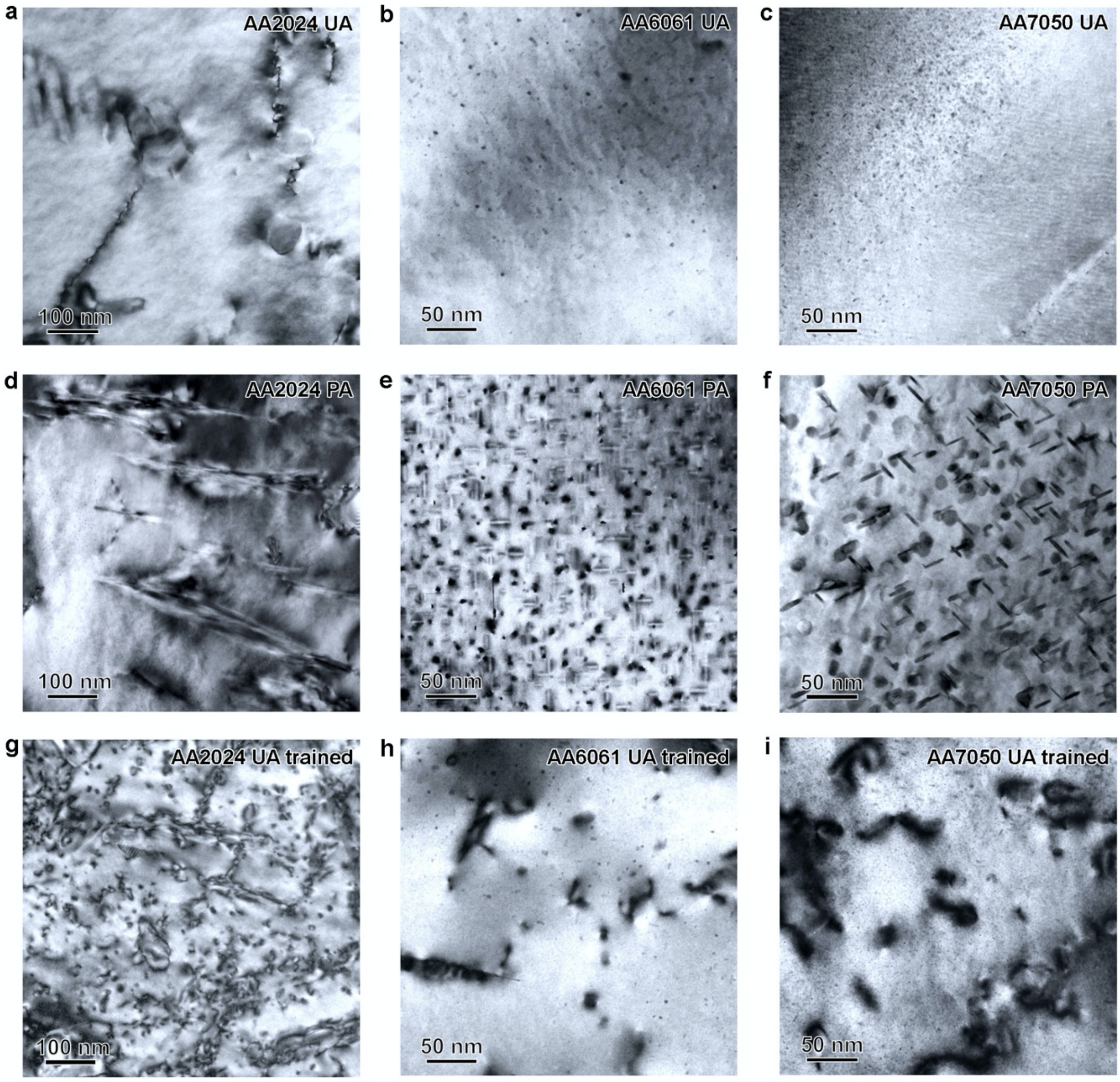

Fig. S2. Bright field TEM images showing initial precipitation microstructure in grains for the under aged (UA) AA2024 (**a**), AA6061 (**b**) and AA7050 (**c**), the peak aged (PA) AA2024 (**d**), AA6061 (**e**) and AA7050 (**f**), and the cyclically trained UA AA2024 (**g**), AA6061 (**h**) and AA7050 (**i**) alloys. The electron beam is closely parallel to $<001>_{Al}$ for AA2024 and AA6061, and is closely parallel to $<110>_{Al}$ for AA7050.

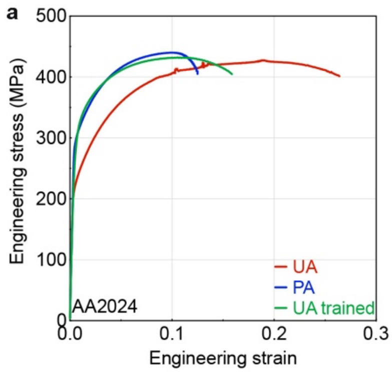

Fig. S3. Engineering stress strain curves for UA, PA and trained AA2024 (**a**), AA6061 (**b**) and AA7050 (**c**) alloys.

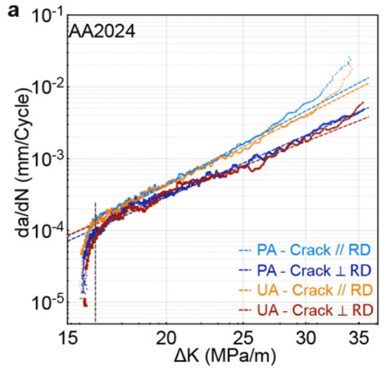

Fig. S4. The fatigue crack growth (FCG) da/dN-ΔK curves in log scale for PA and UA AA2024 (**a**), AA6061 (**b**) and AA7050 (**c**) alloys. Tests were performed with pre-cracks both parallel (//) and perpendicular (⊥) to the rolling direction (RD).

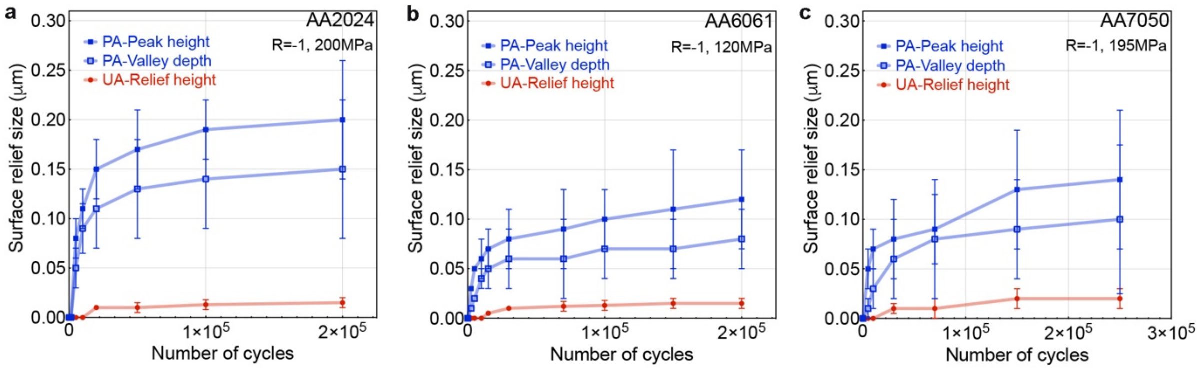

Fig. S5. Summary of the surface relief size evolution for UA and PA samples during fatigue. The stress amplitudes applied are 200 MPa for AA2024 (**a**), 120 MPa for AA6061 (**b**) and 195 MPa for AA7050 (**c**).

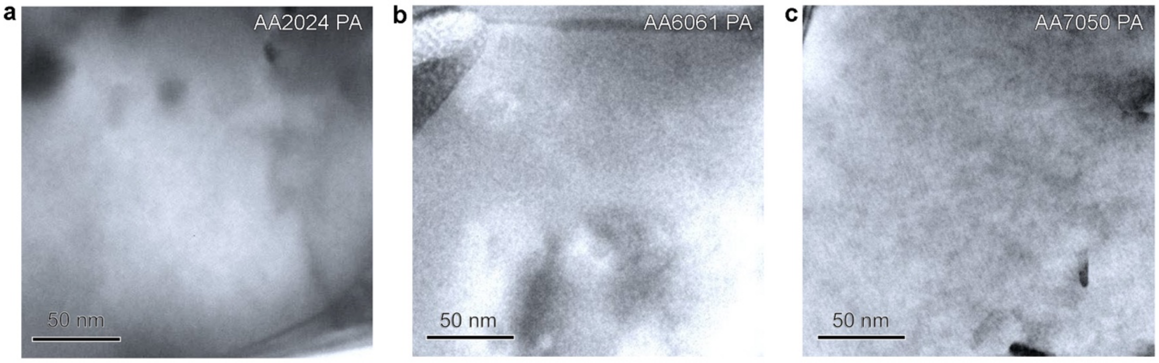

Fig. S6. Bright field TEM images showing the microstructure in PFZ's for PA and HCF treated AA2024 (**a**), AA6061 (**b**) and AA7050 (**c**) alloys. The electron beam is closely parallel to $<001>_{Al}$ for AA2024 and AA6061, and is closely parallel to $<110>_{Al}$ for AA7050. AA2024 was fatigued at 185 MPa for 1×10^6 cycles, AA6061 was fatigued at 120 MPa for 2×10^6 cycles and AA7050 was fatigued at 175 MPa for 2×10^6 cycles.

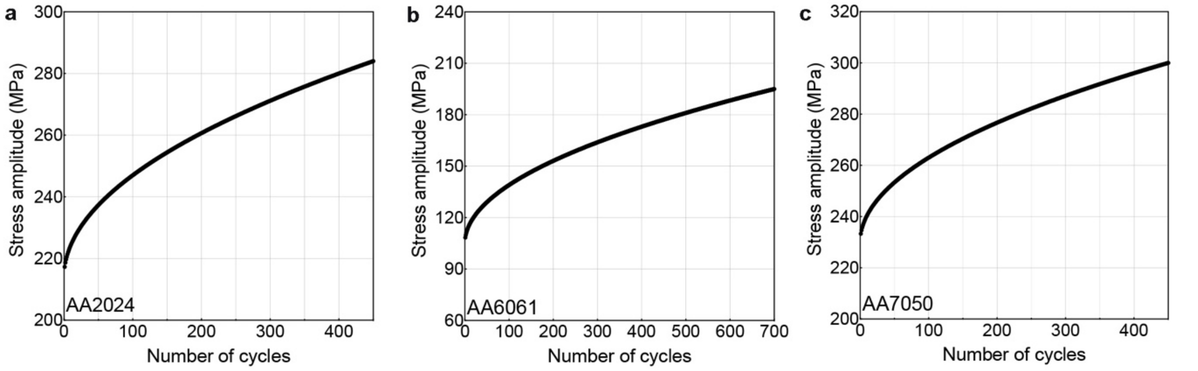

Fig. S7. Cyclic training profiles showing the stress amplitude evolution as a function of cycle number for UA AA2024 (**a**), AA6061 (**b**) and AA7050 (**c**) alloys.

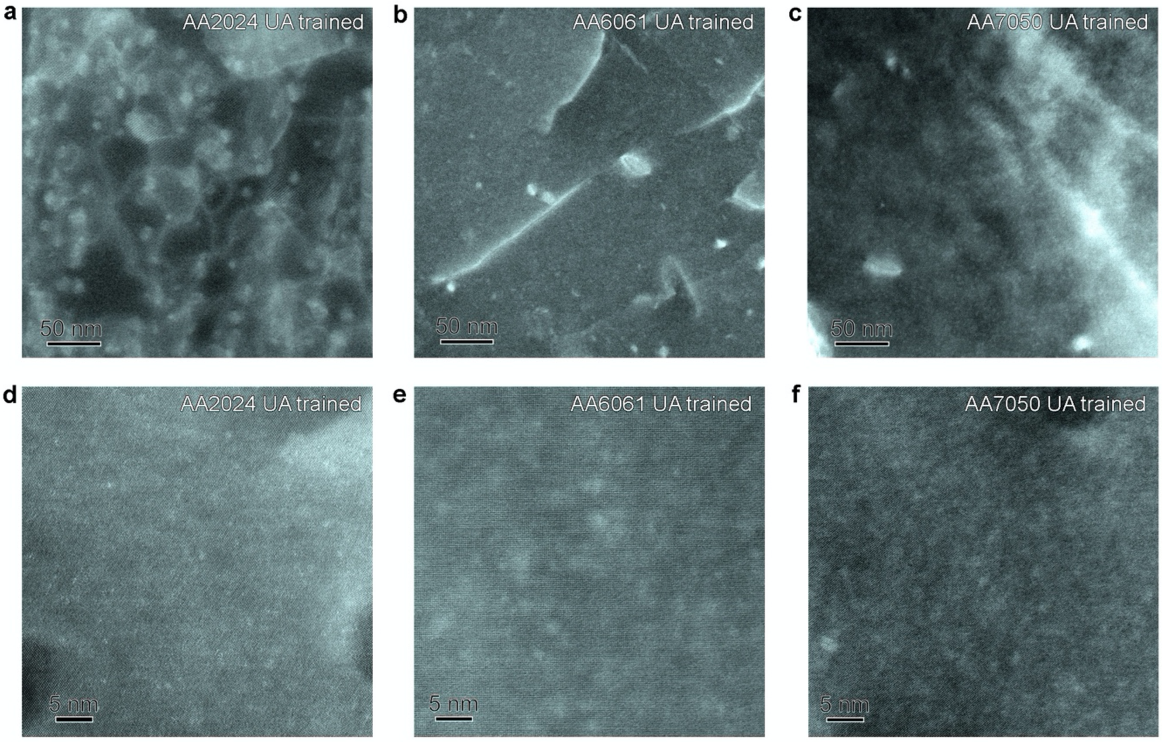

Fig. S8. LAADF-STEM images showing the precipitation microstructure in grains for the cyclically trained UA AA2024 (**a**, **d**), AA6061 (**b**, **e**) and AA7050 (**c**, **f**) alloys. The electron beam direction was parallel to $<100>_{Al}$ for AA2024 and AA6061 and parallel to $<110>_{Al}$ for 7050.

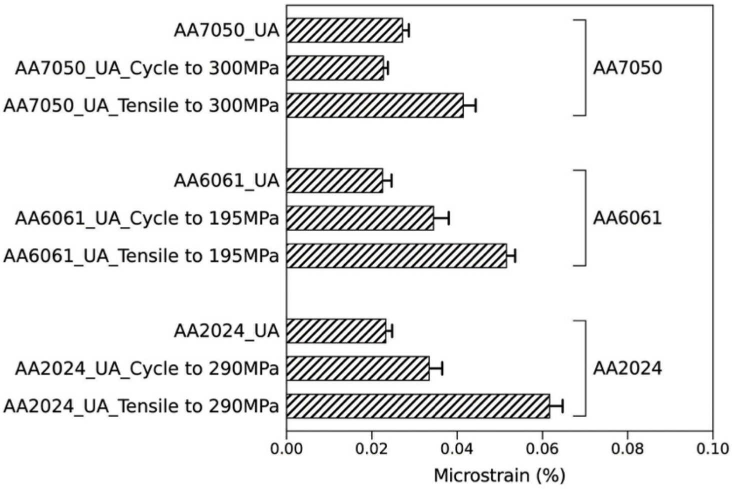

Fig. S9. Comparisons between the microstrains in original UA samples, samples after cyclical training and samples after tensile to the same final training stress (σ_f) for AA2024, AA6061 and AA7050 alloys.
